# Assortative mating for reproductive timing affects population recruitment and resilience in a quantitative genetic model

**DOI:** 10.1111/eva.13524

**Published:** 2023-01-20

**Authors:** Samuel A. May, Jeffrey J. Hard, Michael J. Ford, Kerry A. Naish, Eric J. Ward

**Affiliations:** ^1^ School of Aquatic and Fishery Sciences University of Washington Seattle Washington USA; ^2^ NOAA Fisheries Northwest Fisheries Science Center Seattle Washington USA

**Keywords:** agent‐based model, assortative mating, eco‐evolutionary dynamics, individual‐based model, population structure, quantitative genetics, reproductive success, run timing

## Abstract

Quantitative models that simulate the inheritance and evolution of fitness‐linked traits offer a method for predicting how environmental or anthropogenic perturbations can affect the dynamics of wild populations. Random mating between individuals within populations is a key assumption of many such models used in conservation and management to predict the impacts of proposed management or conservation actions. However, recent evidence suggests that non‐random mating may be underestimated in wild populations and play an important role in diversity‐stability relationships. Here we introduce a novel individual‐based quantitative genetic model that incorporates assortative mating for reproductive timing, a defining attribute of many aggregate breeding species. We demonstrate the utility of this framework by simulating a generalized salmonid lifecycle, varying input parameters, and comparing model outputs to theoretical expectations for several eco‐evolutionary, population dynamic scenarios. Simulations with assortative mating systems resulted in more resilient and productive populations than those that were randomly mating. In accordance with established ecological and evolutionary theory, we also found that decreasing the magnitude of trait correlations, environmental variability, and strength of selection each had a positive effect on population growth. Our model is constructed in a modular framework so that future components can be easily added to address pressing issues such as the effects of supportive breeding, variable age structure, differential selection by sex or age, and fishery interactions on population growth and resilience. With code published in a public Github repository, model outputs may easily be tailored to specific study systems by parameterizing with empirically generated values from long‐term ecological monitoring programs.

## 1 INTRODUCTION

Aggregating species, such as many fishes, sea turtles, and migrating birds, as well as sessile species, such as plants and many benthic invertebrates, have evolved complex and dynamic metapopulations made up of locally adapted subpopulations (Hanski, 1998). This spatial and temporal distribution of individuals can buffer metapopulations against disruptive events that influence any one subunit (Brennan et al., 2019; Schindler et al., 2010). Dynamic processes of dispersal and gene flow between subpopulations can reduce fitness losses due to inbreeding depression (Greenwood et al., 1978), influence rates of adaptation (Garant et al., 2007), and facilitate colonization or recolonization of available habitat (Yeakel et al., 2018). However, an understudied aspect of metapopulation biology is the effect of within‐population eco‐evolutionary processes on population fitness and viability, such as assortative mating or fine‐scale spatial or temporal substructure. Despite the prevalence of such processes in real populations, the majority of existing models used to inform management about the predicted effects of anthropogenic perturbations (e.g., harvest, climate change, and supportive breeding) assume random mating within populations. Disentangling the effects of processes that influence fine‐scale population sub‐substructure or assortative mating is more complicated but will more accurately represent the dynamics of wild populations.

Non‐random mating within a population can occur through a variety of overlapping mechanisms (Crespi, 1989; Kopp et al., 2018; Wang et al., 2019). Here, we consider assortative mating as a positive correlation of genotypes or phenotypes between mated pairs (Jiang et al., 2013). This correlation could occur through mechanisms such as (1) mate choice based on phenotype; (2) fine‐scale temporal or spatial autocorrelation of phenotypes, including cases where space or time are considered as ‘traits’; (3) phenotypic changes over time, resulting in an increase in phenotypic correlations between mates; or (4) by patterns of selection on the pool of potential breeders (Jiang et al., 2013; Kopp et al., 2018; Rios Moura et al., 2021; Sørdalen et al., 2018). Assortative mating based on phenotype has been studied in a number of wild fish species, with the majority of examples around the world focused on the evolution of traits such as size (de Borghezan et al., 2019; Rios Moura et al., 2021; Rueger et al., 2016) or color (Utagawa et al., 2016). In the North Pacific Ocean, long‐term ecological monitoring studies on salmon (e.g., Barnett et al., 2019; Ford et al., 2015; Lin et al., 2017; May, 2022; Peterson et al., 2014) have found many cases of spatial and temporal assortative mating. This assortative mating based on temporal (May, 2022) or spatial (Lin et al., 2017; Peterson et al., 2014) variation in spawning behavior suggests that assortative mating in space and time can have positive effects on fitness and population productivity (May, 2022).

Models exploring dynamic processes of assortative mating or substructure within populations have generated a number of expected demographic or evolutionary outcomes. For example, models have shown that unstructured populations may have a lower overall fitness and recruitment compared to highly structured populations (Nahum et al., 2015). Assortative mating may also affect genetic variation and the magnitude and direction of selection regimes, although these effects may vary depending on the trait in question, selective regime, and mechanism for assortative mating (Bolnick et al., 2009; Bolnick & Kirkpatrick, 2012; Sørdalen et al., 2018). Generally, assortative mating can be expected to increase genetic variance among groups (by allowing for genetic divergence via reduced gene flow) while decreasing genetic variation within groups (by limiting the introduction of new alleles and increasing the homogenizing effects of inbreeding and drift; Crow & Felsenstein, 1968). Subsequently, assortative mating may result in faster responses to selection, due to an overall increase in the magnitude of selection within groups and increased genetic variance available to selection among groups (McBride & Robertson, 1963). Compared to a randomly mating system, assortative mating is expected to result in greater population variance in reproductive fitness (and lower effective population size), because mating pairs closer to local fitness optima should have more offspring than sub‐optimal pairs (Devaux & Lande, 2008). Greater phenotypic or genetic diversity associated with assortative mating may consequently increase population stability and resilience (i.e., through portfolio effects; Schindler et al., 2010). The role of assortative mating in facilitating speciation has also been extensively studied (Dieckmann & Doebeli, 1999; Jiang et al., 2013; Kopp et al., 2018). However, the extent to which such fine‐scale processes may impact recruitment or resilience within populations has yet to be fully investigated in wild populations.

Models built on eco‐evolutionary principles offer a way to examine the relative influence of evolutionary processes within metapopulations on recruitment (Palkovacs & Hendry, 2010). Example applications include studies that have investigated impacts of dispersal between subpopulations on local adaptation and population robustness (e.g., Lin et al., 2017; Yeakel et al., 2018), examined effects of harvest on evolution of size or age (Bromaghin et al., 2011; Eldridge et al., 2010), detected the effects of harvest selection on dispersal (Moland et al., 2019) or mate selection (Sørdalen et al., 2018), and studies exploring evolutionary responses to climate change (Reed et al., 2011). A major assumption of many such models is that mating occurs randomly within populations (Bürger, 1999; Gomulkiewicz & Holt, 1995; Kopp & Matuszewski, 2014; Lande & Shannon, 1996), which may bias the interpretation of model outputs if mating systems are assortative in study populations (Godineau et al., 2022; Rios Moura et al., 2021; Rolán‐Alvarez et al., 2015). For example, unaccounted assortative mating may inflate quantitative estimates of additive genetic variance in traits of interest and result in a failure to detect other sources of variation, such as genetic dominance or environmental effects (Vinkhuyzen et al., 2012). Indeed, many empirical studies under‐estimate or fail to detect the extent of assortative mating due to incomplete sampling or pooling of data which represent spatially or temporally heterogeneous trait distributions (Rios Moura et al., 2021; Rolán‐Alvarez et al., 2015). Furthermore, most quantitative genetic models investigating the effects of assortative mating on evolutionary processes (i.e., the effects of selection or gene flow on genetic variation) assume constant or infinite population sizes, and therefore do not incorporate the effects of demography (i.e., changes in population size or density) on evolutionary outcomes (Devaux & Lande, 2008; Felsenstein, 1981; Fisher, 1918; Godineau et al., 2022; Wright, 1921). Recent evidence has shown how demography, which is often directly influenced by anthropogenic perturbations, can have important feedback effects on selective regimes and evolution (Govaert et al., 2019). Thus, an eco‐evolutionary modeling framework that incorporates both assortative mating and demographic dynamics in spatially or temporally heterogeneous populations would facilitate a greater theoretical understanding of anthropogenic effects on wild population dynamics such as recruitment, demographic changes, or the evolution of fitness‐linked traits. Here, we aim to develop such a model.

Previous work has illustrated relationships between genetic or ecological variation and demographic or evolutionary outcomes. Results from similar simulation models are useful for forming hypotheses about the expected behavior of our model. For example, models investigating additive genetic correlations between fitness‐linked traits have shown how genetic correlations can affect the direction and magnitude of evolutionary responses, possibly limiting the evolution of negatively genetically correlated traits to fitness peaks and reducing population mean fitness as a result (Duputié et al., 2012; Etterson & Shaw, 2001; Via & Lande, 1985). Such antagonistic correlations are particularly common in cases of genetically correlated traits under variable sexual selection (Connallon & Hall, 2016). Recent simulation efforts have also examined how shifts in phenotypic optima over time or increases in environmental variability (i.e., due to climate change) can cause a lag between population mean phenotypes and fitness optima (Cotto et al., 2019; Godineau et al., 2022; Reed et al., 2011). These studies have demonstrated how increased environmental variation can lead to reductions in mean fitness and subsequent demographic declines, with possible eco‐evolutionary feedbacks to adaptive processes (Cotto et al., 2019). Such dynamics can be further complicated by variation in the direction or magnitude of local selection regimes, which are typically modeled as Gaussian stabilizing selection curves that vary among generations. For example, populations exposed to the combined effects of both fluctuating ecological optima and intensifying selection may experience a larger lag (i.e., lag load) between population mean phenotypes and ecological optima than with either dynamic alone (Hellmann & Pineda‐Krch, 2007), and dramatic demographic declines may follow (Bürger & Krall, 2004). Examination of these types of combined effects can be particularly difficult to parse apart in empirical studies but are amenable to study through simulation‐based models. Thus, it is prudent to incorporate dynamics such as genetic correlations, environmental variability, and variable selection intensity in quantitative genetic models seeking to comprehensively examine population dynamics.

The objectives of our paper are to first develop a modular and flexible framework for simulating the demographic and evolutionary impacts of different mating types (assortative or random) in variable environments and under variable strengths of multivariate selection, and second to conduct a sensitivity analysis of the relative effects of each input parameter on evolutionary and demographic outcomes (i.e., ensuring that model behavior follows established theory). Parameters in this model could be informed by empirical observations or drawn from the literature. This model might then be used in a management setting to understand the consequences of changes in parameters for future recruitment, population growth rate, and trait evolution. We next demonstrate the utility of this model across a series of four in silico experiments, focusing on the trait of reproductive phenology within a typical salmonid life history pattern. Pacific salmon are characterized by an anadromous, semelparous life‐history in which adult salmon return from a period of ocean residence to spawn for a brief period in their natal streams and then die (Quinn, 2018). This life history means that a single breeding event represents an individual's total contribution to the next generation, so mate availability and reproductive timing are of particular importance to recruitment in salmon populations. Empirically, the time period and duration of return to freshwater spawning grounds (‘run timing’) varies among and within species and populations and has been shown to be adaptive to the environmental conditions in specific rivers (reviewed by Quinn, 2018; Waples et al., 2022). A natural consequence of variation in run timing within a population, when combined with a short duration of reproductive behavior, is some degree of positive assortative mating on return time. Gaining a better understanding of how such assortative mating on return time influences population viability and productivity is therefore clearly of interest to conservation and fishery management of salmon (Waples et al., 2022). To help fill this need, we evaluate how sensitivity analyses of (1) assortative mating, (2) correlation between multiple temporally based fitness‐linked traits, (3) environmental variation, and (4) the strength of selection affect changes in population size (census, effective) and trait evolution. All code and data are provided in our publicly available Github repository linked here: https://github.com/SMay1/Assortative_Mating_QG_IBM

## 2 METHODS

### 2.1 Model overview and assumptions

We designed an individual‐based modeling framework capable of predicting trait values (i.e., phenotypes) and fitness for all individuals in a population over short, contemporary evolution timescales (i.e., generation over generation). While our focus is on parameters relevant for salmonids, this framework is general and can be extended to other species. For example, fitness was quantified as individual lifetime reproductive success: the number of adult offspring produced by each adult individual, and the size of the subsequent generation was assumed to be the sum of these realizations of reproductive success across individuals. However, the fitness module could easily be extended to incorporate fecundity, survival, or growth (as in Lin et al., 2017), if different fitness metrics are desired for other study systems. We also assumed selection acted on traits with constant phenotypic and additive genetic variances to predict individual fitness values, although, phenotypic variance could be made to vary in future applications. Offspring were assigned trait values, following quantitative genetic theory for deterministic inheritance (i.e., alleles were not modeled; Roff, 2012). At each time step, trait and fitness values for individuals within generations were used to quantify population‐level summary statistics such as census population size (Nc), effective population size (Ne), and the mean and variance of phenotypic traits; although, additional summary statistics may also be estimated, such as the per‐capita population growth rate (*λ*) or geometric mean fitness.

To demonstrate the utility of this type of mechanistic model for visualizing and predicting changes to vital population parameters, we utilized a range of known input parameter values and population dynamics from pedigreed populations of anadromous, semelparous salmonids (Lin et al., 2017; May, 2022; Quinn et al., 2007). Specifically, we explored two phenological traits of interest: return day and reproductive lifespan (RLS). Return day was defined as the calendar day of the spawning season individuals returned to spawn. RLS was defined as the number of days individuals survived on the spawning grounds after arrival. Return timing and RLS are important traits in salmonid populations, as they are closely linked to fitness (Evans et al., 2019; Koch & Narum, 2021; Lin et al., 2016). Selection typically acts strongly on salmonid phenological traits, as fitness is closely linked to seasonal ecological dynamics (i.e., egg incubation temperatures and food availability for emerging fry), and there is a high potential for phenotype‐environment mismatch if fitness is closely tied to environmental conditions at the time of stream entry (Evans et al., 2019). Longer RLS values are associated with greater reproductive opportunity and ultimate reproductive success; although there likely exists an energetic tradeoff associated with gamete production, mating opportunity, defense against redd superimposition, and predation risk (Hendry et al., 2004; Lin et al., 2016). These two traits determine possible mating pairs within populations, as two fish may only mate if they are on the spawning grounds at the same time. In addition, return day and RLS have been shown to be inversely correlated, both phenotypically and genetically, in many salmon populations, where early returning fish tend to have longer RLS than late returns (Hendry et al., 2004; Lin et al., 2016; McMahon, 2021). Significant heritability values have been attributed to both traits (Carlson & Seamons, 2008; Lin et al., 2016). We describe our model in the context of these population dynamics, where within each generation: (1) individuals return to spawning grounds on a specific day and are able to mate for a specific number of days, depending on their reproductive lifespan (reproduction); (2) each mating pair is assigned a number of offspring, depending on the deviance of their trait values from optimal return timing and RLS values (selection); and (3) individual offspring inherit trait values from their parents, deviating according to a constant phenotypic trait variance and covariance in the population. Subsequently, offspring become reproductive adults in the following generation, and ecological optima vary according to a predefined intergenerational environmental variance.

To simplify model mechanics and to focus on parameters of interest, we simplified some aspects of salmonid life history. Specifically, we assumed discrete generations, which is not typical for most salmonid species, where age‐at‐maturity can vary (Quinn, 2018). We also assumed a constant and equal adult sex ratio of 1:1, which can in fact vary in natural populations (Brykov et al., 2008; Quinn et al., 1996). The duration of the spawning season (in days) was held constant over time, whereas in natural populations the length of the spawning season may vary among generations (although generally consistent within a given population). Similarly, the phenotypic variance and covariance between quantitative traits was held constant over generations, which is a common assumption of quantitative genetic models when population sizes are sufficiently large, selection is sufficiently weak, or evolutionary timescales are relatively short (Roff & Mousseau, 2005). We also assume that our simulated populations were not affected by additional intrinsic or extrinsic factors, including carrying capacity, density regulation, predation or fisheries harvest, dispersal, nor inbreeding depression, which can be important regulatory dynamics in real populations (Koch & Narum, 2021). Males and females in our model were assumed to mate at the same frequency; however, mating frequency can differ substantially between male and female salmon depending on population‐specific sex ratios and species‐specific mating behavior (Bentzen et al., 2001). Similarly, males are known to return to streams earlier than females in many salmon species, yet our model assumes no difference in return timing between the sexes. Lastly, we assume that the quantitative traits examined here are controlled by many loci of small effects (infinitesimal model). Some recent studies have shown how a large proportion of phenotypic variation in run timing is influenced by a gene‐region of large effect in some salmon species, along with additional loci of smaller effect (reviewed by Waples et al., 2022). We describe our model and frame our conclusions from our experiments with these assumptions in mind.

### 2.2 Model initialization

We assumed that the duration of the spawning season for our model was a 30‐day window, during which time individuals returned to spawning streams to mate and reproduce. This length of spawning season is similar to many small stream‐spawning populations of Pacific salmon (Lin et al., 2016; Quinn et al., 2007), but may not encompass some cases of very early ‘premature’ migration seen in some salmon species (Quinn et al., 2016; Waples et al., 2022). To initialize the first generation (F0) of individuals and their trait values, the following values were input as initial model parameters (values provided in Table 1): an initial population size (Nc_initial_), population means for return day and RLS (*μ*), population variance in these traits (*σ* ^2^), and a correlation coefficient between traits (*ρ*). Values for the phenotypic variance–covariance matrix of trait values (**P**) were quantified from *σ* ^2^ and *ρ*. Individuals were randomly assigned as either males or females, with equal probability. Phenotypic traits (return day, RLS) for each individual were drawn from a truncated bivariate normal distribution, where the values of each trait ranged from 1–30 days. To ensure RLS values did not exceed the 30‐day spawning season, RLS values were conditioned on return day such that RLS could not be greater than the difference between the length of the spawning season and the day of an individual's return. Future applications of this framework to other traits of interest may choose not to apply a truncated distribution, which should generate similar results if phenotypic variances are sufficiently small or selection is sufficiently strong. Number of generations was also set an initial input parameter; all simulations included in this study were set to iterate for 10 generations, as all chosen input parameters (provided in Table 1) and model assumptions (e.g., constant phenotypic variance and deterministic inheritance) resulted in rapid evolution of traits to equilibria and clearly apparent trends in population growth.

**TABLE 1:** Symbols and descriptions used in the text to describe model parameters.

Category	Parameter	Values used	Description
Input parameters	Nc_initial_	500	Census population size of founder generation (F0)
	*μ*_Return Day_	10	Initial population mean day of return to spawning grounds
	*μ*_RLS_	5	Initial population mean reproductive lifespan after returning to spawn
	*σ* ^2^_Return Day_	10, 20, 30	Population phenotypic variance in return day
	*σ* ^2^_RLS_	20	Population phenotypic variance in reproductive lifespan
	⍴	−0.6, −0.3, 0	Phenotypic correlation between return day and reproductive lifespan; ranges from −1 to 1
	**ω**	1, 2, 3	Strength of selection (1/**ω**), measured as a scalar: the number of phenotypic standard deviations in a stabilizing selection curve
	*σ* ^2^_θ Return Day_	10, 20, 30	Interannual environmental variance in optimal return day
	*σ* ^2^_θ RLS_	1	Interannual environmental variance in optimal reproductive lifespan
	$\bar{\theta }$	[*μ*_Return Day_, *μ*_RLS_]	A vector of mean phenotypic optima
Internal parameters	θ_Return Day_	*μ*_Return Day_ +/− *σ* ^2^_Return Day_	Phenotypic optimum for return day within a given generation
	θ_RLS_	*μ*_RLS_ +/− *σ* ^2^_RLS_	Phenotypic optimum for RLS within a given generation
	**P**	**G + R**	**P**, **G**, and **R** represent the phenotypic, additive genetic, and residual (i.e., environmental) variance–covariance matrices, respectively. They are all assumed constant, and P is estimated directly from ***μ***, ***σ*** ^**2**^, and ⍴
	**θ**	[θ_Return Day_, θ_RLS_]	A vector comprising θ_Return Day_ and θ_RLS_ within a given generation
	*W*(z)	Equation 1	Expected fitness weight from trait values alone; ranges from zero to one
	RS_exp_	qpois(*W*(z))	Expected lifetime reproductive success from trait values alone, drawn from a Poisson distribution with *λ* = 2 and a probability weight = *W*(z)
	*W*_i,j_	Equation 2	The weight of male *i* and female *j* being assigned offspring from a pool of potential offspring, determined from the product of their relative RS_exp_ values and whether they overlapped in time [1] or not [0]
	RS_obs_	∑offspring _i,j_	The observed number of offspring assigned to each individual or mated pair
Output parameters	Nc		Census population size of a cohort
	Ne		Inbreeding effective population size of a cohort (as in Waples & Waples, 2011)
	$\bar{\text{Return}\;\mathrm{Day}}$		Mean return day within a cohort
	$\bar{\mathrm{RLS}}$		Mean reproductive lifespan within a cohort

*Note*: All values used for input parameters in the main text are provided, in addition to shorthand equations for how internal parameters were quantified.

### 2.3 Fitness estimation module

Stabilizing selection was applied to potential parents to generate expected fitness values from individual traits, using methods adapted from Lin et al. (2017). Expected fitness estimation selected against individuals with trait values that deviated from population optima for return timing and RLS. Individuals were assigned an expected fitness weight using the following equation (adapted from Lande, 1979; see also Lin et al., 2017):

(1) $$W\left(z\right)=\exp\left\{-\frac{1}{2y}{\left(z-\theta \right)}^{T}\omega^{-1}\left(z-\theta \right)\right\}$$

where y is a scaling factor that scales individual fitness weights, *W*(z), to a minimum of zero and a maximum of one; z is a vector of individual trait values; **θ** is a vector of trait optima (θ_RLS_ and θ_Return Day_); and **ω** is a matrix describing the shape of the fitness landscape (Lin et al., 2017). This multivariate model is equivalent to a bivariate Gaussian distribution with mean **θ** and covariance matrix **ω**. The off‐diagonal elements of **ω** (which are identical) represent the correlation between entry day and reproductive lifespan, and the diagonal elements represent the magnitudes of variation for each of these traits; together these parameters control the shape of the fitness landscape. We assumed that the strength of stabilizing selection was equal for the two traits and constant across generations. To simulate interannual variation in local selection regimes, trait optima were allowed to vary each generation according to a Gaussian distribution with a mean equal to the initial phenotypic means and a variance vector ***σ*** ^**2**^_**θ**_, comprising *σ* ^2^_θ RLS_ and *σ* ^2^_θ Return Day_ set as an initial input parameter.

Next, we quantified individual expected reproductive success values (RS_exp_), defined as the expected number of offspring per adult breeder, according to only their phenotypic trait values for return day and RLS. To quantify RS_exp_, individual fitness weights (*W*(z)) were used as probabilities to draw from a Poisson distribution with *λ* = 2, representing a stable population.

### 2.4 Reproduction module

The census size of the next generation was determined by summing the RS_exp_ values for all females in the population, as male salmon are able to spawn with multiple females and are therefore generally not considered to limit reproduction. Individuals from this pool of offspring were assigned a dam and sire in the parental generation. Assortative mating for return timing was incorporated into this model through a weighted draw of offspring for possible parent pairs, using adapted methods from *TheWeight* (Waples, 2022):

(2) $$W_{i,j}=W_{\mathrm{mi}}\times W_{\mathrm{fj}}\times W_{o\;i,j}=\frac{{\mathrm{RS}}_{\exp\;i}}{\bar{{\mathrm{RS}}_{\exp}}}\times \frac{{\mathrm{RS}}_{\exp\;j}}{\bar{{\mathrm{RS}}_{\exp}}}\times \left[1,0\right]$$

where the weight ($W_{i,j}$) of male *i* and female *j* being assigned a given offspring from the pool of potential offspring was determined by the product of three probabilities: (1) the relative fitness of male *i* ($W_{\mathrm{mi}}=\frac{{\mathrm{RS}}_{\exp\;i}}{\bar{{\mathrm{RS}}_{\exp}}}$), (2) the relative fitness of female *j* ($W_{\mathrm{fj}}=\frac{{\mathrm{RS}}_{\exp\;j}}{\bar{{\mathrm{RS}}_{\exp}}}$), and (3) an overlap weight ($W_{o\;i,j}=\left[1,0\right]$). The overlap weight was a binomial probability, where if male *i* and female *j* were present in the stream at the same time during the spawning season (overlapped) the pair was given an overlap weight of one. If male *i* and female *j* did not overlap, the pair were given an overlap weight of zero, resulting in an overall probability equal to zero that pair *i,j* were assigned offspring ($W_{i,j}=0$). Thus, the mechanisms by which assortative mating acted here was through both mate choice and selection, where mate choice was defined as the opportunity to mate only between individuals on the spawning grounds at the same time. Overlap weights did not give higher probability of mating to pairs who overlapped for many days, relative to pairs who only overlapped for a short while; however, this dynamic may be incorporated in future model applications.

To model random mating, *W*_*o i,j*_ was set equal to one, such that all possible mating pair combinations of male *i* and female *j* had an equal probability of mating, and mate availability was not causing assortative mating. However, assortative mating still acted to some extent via selection, because *W*_*mi*_ × *W*_*fj*_ caused mating pairs close to the optimum to be assigned more offspring than sub‐optimal pairs. Retaining *W*_*mi*_ × *W*_*fj*_ while setting *W*_*o i,j*_ equal to one provided a mechanism that allowed for evolution under random mating with no assortative mating due to mate choice. In contrast, some quantitative genetic models allow for selection without explicitly modeling mate choice (e.g., Ford, 2002). Future applications of this model may consider modifying Equation 2 to allow or disallow for different mechanisms that result in assortative mating. For example, setting both *W*_*mi*_ and *W*_*fj*_ equal to one while retaining *W*_*o i,j*_ would remove assortative mating via selection but allow assortative mating via mate choice. Alternatively, setting *W*_*mi*_ * *W*_*fj*_ and *W*_*o i,j*_ equal to one would provide a mechanism to examine a randomly mating system with no evolution. In this way, our framework allows flexibility to isolate specific mechanisms and alter the model to fit specific study systems.

The final number of offspring assigned to each parent defined that parent's observed reproductive success (RS_obs_), which was later used to quantify output parameters.

### 2.5 Inheritance module

**FIGURE 1:**
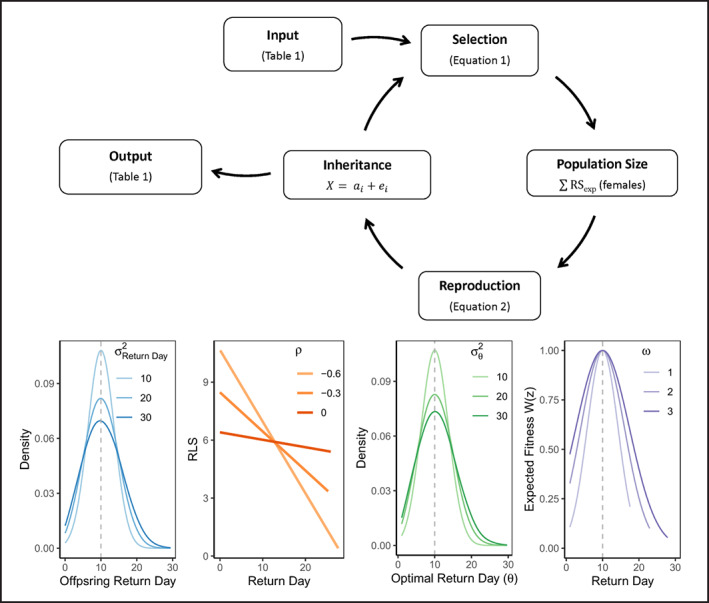
Schematic of model workflow (top) and visualization of univariate parameter space in four simulation experiments (bottom). Experiment 1 (blue) shows how increasing phenotypic variance in return day (*σ* ^2^_Return Day_) increases the likelihood that an offspring's trait value will deviate from their mid‐parent value (dashed gray line). Experiment 2 (orange) shows the phenotypic relationship between return day and reproductive lifespan under different phenotypic correlations (⍴). Experiment 3 (green) shows how the optimal return day (θ_Return Day_) for a given cohort is more likely to deviate from the mean ($\bar{\theta }$_Return Day_) when variance (*σ* ^2^_θ Return Day_) is high. Experiment 4 (purple) gives expected fitness values associated with return day, for three different strengths of selection regime (**ω**, quantified using Equation 1).

Offspring were assigned trait values following established quantitative genetic theory on deterministic inheritance of correlated traits (i.e., alleles were not explicitly modeled; Roff, 2012). Phenotypic variation in a trait (X) is equal to the sum of its genetic (G) and environmental (E) components of variation. Thus, to simulate inheritance of a univariate trait, an offspring's trait value may be drawn from a Gaussian distribution with a mean equal to the mid‐parent value and a variance equal to the phenotypic variance in the population (X = G + E). Incorporating correlations between multiple traits, this concept may be extended: the phenotypic covariance matrix (**P**) is equal to the sum of the genetic covariance matrix (**G**) and a covariance matrix of residual deviation (**R**). Thus, for our correlated traits of return day and RLS, we simulated trait values for individual offspring from a truncated bivariate normal distribution. The means of this distribution were equal to the mid‐parent values of the two traits, and the multivariate variance and covariance of the two traits were obtained from the phenotypic covariance matrix (**P** = **G** + **R**), an initial input parameter where **P**, **G**, and **R** were assumed constant across generations (Figure 1, Experiment 1). As in the initialization of traits for the F0 generation, individual values for RLS were conditional on individual values of return day, such that RLS could not be greater than the length of the spawning season minus the return day. This prevented individuals from surviving and breeding past the last day of the spawning season. Once offspring were assigned traits, they became the parents of the next generation, and the model repeated for a set number of generations.

### 2.6 Model outputs

Model outputs comprised a full pedigree (animal, dam, sire), trait values, and RS_obs_ values for each individual in the population. These data were then used to quantify population statistics within generations. Here, we estimated the mean and variance of trait values, census population size (Nc), and effective population size (Ne); however, many other output parameters could be estimated (i.e., population growth rate or operational sex ratios). Effective population size was quantified as the ‘inbreeding effective size’ following the methods of Waples and Waples (2011), which incorporates the population mean and variance in reproductive success. We computed a mean and 95% confidence interval from replicated simulations for each model output, within generations. By varying input parameters or changing model dynamics, changes in these population statistics may be directly observed.

### 2.7 Sensitivity analyses

To validate that model outputs conformed to expected theoretical outcomes for multivariate selection on correlated traits, we varied model input parameters to address four eco‐evolutionary questions. Each unique combination of input parameters was considered a simulation, which were each projected for 10 generations. For most parameter combinations, the first 3–4 generations of a particular iteration represented a ‘burn‐in’ period. To capture variation in model outputs for each set of input parameters, we performed 100 iterations per simulation (e.g., initializing and projecting 100 populations). If the population size in a given simulation dropped below 50, we considered that population functionally extinct. An assortative mating system was assumed, except in Experiment 1 where assortative and random mating systems were compared. For all simulations in this study the following input parameters were held constant at Nc_initial_ = 500 individuals, *μ*_Return Day_ = 10 days, *μ*_RLS_ = 5 days, *σ* ^2^_RLS_ = 20 days, and *σ* ^2^_θ RLS_ = 1 (See Table 1 for parameter definitions). We iteratively varied each of the following input parameters, which were otherwise held constant at intermediate values of *σ* ^2^_Return Day_ = 20 days, *ρ* = −0.3, *σ* ^2^_θ Return Day_ = 20 days, and **ω** = 2 phenotypic standard deviations.

#### 2.7.1 Experiment 1: Assortative mating

The effects of temporal assortative mating on population dynamics were examined by varying phenotypic variance in return day across 3 levels (10, 20 and 30 days) to represent strong, moderate, and weak assortative mating (Figure 1, Experiment 1). Conceptually, variance in return day affects assortative mating through its relationship with heritability: lower variance increases the probability that individuals will return closer to when their parents returned (i.e., greater heritability of return day). In turn, greater heritability increases the mating probability among individuals with similar trait values (i.e., assortative mating). However, phenotypic variance also affects fitness: greater phenotypic variance is expected to result in greater RS_exp_ for a given trait value, because the strength of selection is quantified in phenotypic standard deviations (Equation 1). We expand on this phenomenon in the Figure S1 and interpret results of this simulation with these dynamics in mind. Within this framework, we also compared simulations with assortative mating (detailed in the reproduction module) to random mating where dam‐sire‐offspring triads were assigned randomly (*W*_*i,j*_ = 1 for all putative pairs).

#### 2.7.2 Experiment 2: Trait correlation

The effect of correlation between traits on population dynamics was examined by varying the magnitude of phenotypic correlation (*ρ*) between return day and RLS (values of −0.6, −0.3, and 0 to represent strong, moderate, and weak/no correlation between traits; Figure 1, Experiment 2). A negative correlation between return timing and RLS has been consistently reported in Pacific salmon populations, suggesting that early returning individuals tend to live longer on spawning grounds than later returning individuals (Doctor & Quinn, 2009; Lin et al., 2016; McMahon, 2021). Doctor and Quinn (2009) suggested that these correlations may indicate evidence for adaptive differentiation associated with temporal isolation (i.e., adaptation‐by‐time; Hendry & Day, 2005). Lin et al. (2016) reported significant heritability values, phenotypic correlations, and genetic correlations between RLS and return day, indicating that these traits are likely co‐inherited. The correlation values used here (0 to −0.6) approximately represent the range of published correlation values for these two traits (−0.3 to −0.5). We additionally explored the effect of positive correlations on simulation outputs in the Figure S2, although we note that positive correlations between RLS and return timing are not biologically relevant in semelparous salmonid populations.

#### 2.7.3 Experiment 3: Environmental variability

We examined the effect of environmental variability on demography by adjusting the magnitude of interannual variation in ecological trait optima (*σ* ^**2**^_**θ**_). Separate simulations were run for *σ* ^2^_θ Return Day_ values of 10, 20, and 30 days to represent temporally stable, slightly variable, and extremely variable local selection regimes (Figure 1, Experiment 3). In effect, we examined population responses to fluctuating selection associated with increased environmental variability, one key prediction of climate change studies. Varying *σ* ^2^_θ RLS_ should result in identical fitness outcomes as *σ* ^2^_θ Return Day_, but the fitness effects of simultaneous movement of both optima may prove difficult to interpret. Therefore, we chose to isolate the effects of *σ* ^2^_θ Return Day_ by holding *σ* ^2^_θ RLS_ constant at a value of 1, representing extremely low variation in optimal RLS. This approach is similar to many evolutionary models used to predict evolutionary responses to climate change scenarios (Bürger, 1999; Gomulkiewicz & Holt, 1995; Kopp & Matuszewski, 2014; Lande & Shannon, 1996). Some such models quantify specific environmental variables separately (i.e., temperature, flow; Reed et al., 2011). Here, we instead consider all sources of inter‐annual environmental variability contributing to return timing selection regimes as a single variable. We also note that future model applications to climate change scenarios may also consider inducing a trend in the mean trait optima ($\bar{\theta }$), which is likely to co‐occur with increased environmental variation. An example simulation where variance in phenotypic optima is null is provided in the Figure S3.

#### 2.7.4 Experiment 4: Strength of selection

The effect of the strength of selection on population dynamics was examined by varying the diagonal elements of **ω** (Equation 1). Conceptually, the diagonal elements of **ω** correspond to the number of phenotypic standard deviations in the stabilizing selection curve for each trait (Hard, 2004). The strength of selection on the two traits, typically expressed as 1/**ω**, was assumed equal. We adapted the methods of Hard (2004) by examining selection intensities ranging from very strong (1 standard deviation) to weak (3 standard deviations; Figure 1, Experiment 4), according to previously published estimates (Guillaume & Whitlock, 2007; Jones et al., 2003; Lin et al., 2017). Hard (2004) notes that empirical evidence indicates that natural selection (stabilizing and directional) is generally weak in natural populations (3–4 standard deviations) but might be stronger (1–2 standard deviations) in harvested populations (Kingsolver et al., 2001). The shape of the stabilizing selection curve (**ω**) is related to the quadratic selection gradient (**γ**) approximately by **γ** = −1/**ω** ^2^ (Arnold et al., 2001). Thus, the range of **ω** used here (1–3 standard deviations) should encompass most moderate to strong selection scenarios reported by Kingsolver et al. (2001), particularly in harvested populations (Hard, 2004). Selection was modeled as stronger than in some natural populations to prevent exponential population growth, as no other regulatory dynamics acted in these scenarios (i.e., predation or density dependence); although, such regulatory dynamics may easily be incorporated in future model applications.

## 3 RESULTS

### 3.1 Experiment 1: Assortative mating

**FIGURE 2:**
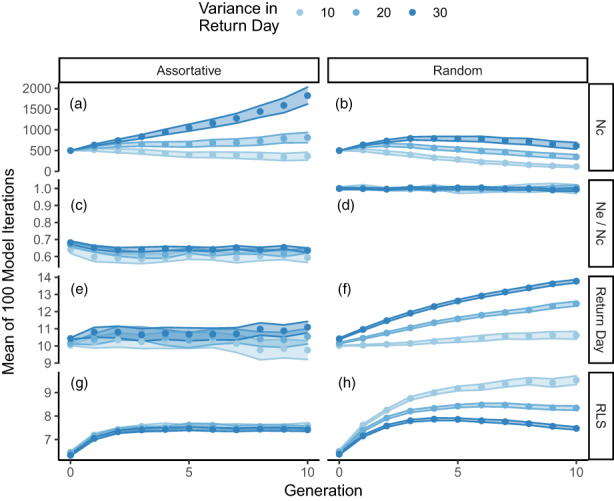
Demographic output parameters (*y*‐axes) over 10 generations (*x*‐axes) for three simulations with variable input parameter values for phenotypic variance in return day (*σ* ^2^_Return Day_; color shades). The mating system within simulations was either assortative (left) or random (right). Output parameters of interest (points), from top to bottom, were census population size (Nc), ratio of effective to census population size (Nc/Ne), mean return day, and mean reproductive lifespan. Output parameters were estimated as the mean of 100 model iterations and are bounded by 95% confidence intervals.

Demographic outcomes differed between simulations with assortative and random mating systems. In simulations with equal variance in return day (*σ* ^2^_Return Day_), assortative mating generally translated into larger population sizes than simulations with random mating. For example, in simulations where the variance in return day was intermediate (20 days), the mean population size with random mating was 348 individuals, 95% CI [294–402] after 10 generations, but under assortative mating this value was 813 individuals, 95% CI [689, 937] (Figure 2a,b). From this result we concluded that assortative mating systems contributed to population growth, whereas random mating systems limited recruitment. We further examined the relationship between *σ* ^2^_Return Day_ and population size (Nc) and found a positive relationship under both random and assortative mating: greater phenotypic variance resulted in higher recruitment (see Figure S1 for additional details on the relationship between phenotypic variance and fitness). However, this relationship was approximately linear under random mating and nonlinear under assortative mating (Figure 2a,b; Figure S4), as populations grew much faster with increasing *σ* ^2^_Return Day_ under assortative mating than under random mating.

Evolutionary outcomes also differed between scenarios with random and assortative mating. Under random mating, the effective population size of all scenarios was approximately equal to the census size (Ne/Nc = 1; Figure 2c,d). This result was expected, as random mating (the equal probability of adults to produce offspring) is a key attribute of the Wright‐Fisher Idealized Population Model upon which Ne is contingent. Furthermore, the ratio between Ne and Nc under assortative mating was positively correlated with *σ* ^2^_Return Day_, corresponding to slightly larger Ne/Nc ratios when *σ* ^2^_Return Day_ was high. Investigation of the evolution of return day and RLS revealed that the distribution of these traits depended greatly on phenotypic variance under random mating: *σ* ^2^_Return Day_ was positively correlated with mean return day (Figure 2e,f) but negatively correlated with mean RLS (Figure 2g,h). On the other hand, trait evolution was mostly independent of phenotypic variation under assortative mating, although greater *σ* ^2^_Return Day_ resulted in slightly higher mean return day (Figure S5).

### 3.2 Experiment 2: Trait correlations

**FIGURE 3:**
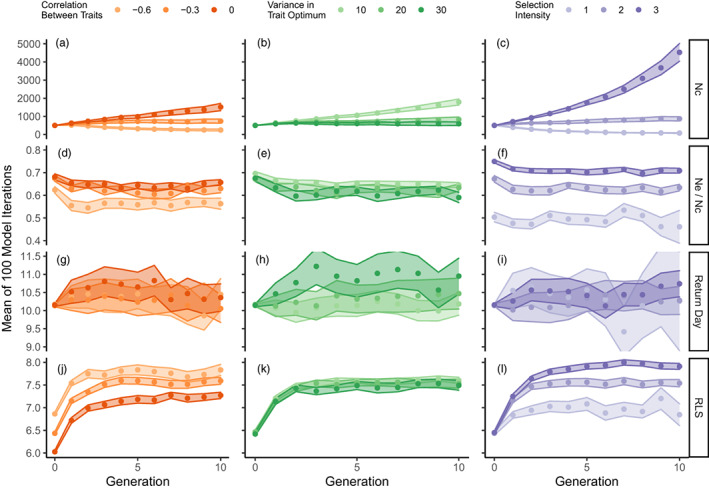
Demographic output parameters (*y*‐axes) over 10 generations (*x*‐axes) for three simulation experiments (colors). Each experiment consisted of three variations of a single input parameter (shades), while all other parameters were constant under an assortative mating system. The input parameters of interest were phenotypic correlation between return day and reproductive lifespan (⍴; oranges), variance in optimum return day (*σ* ^2^_θ Return Day_; greens), and strength of selection regime (**ω**; purples). Output parameters of interest (points), from top to bottom, were census population size (Nc), ratio of effective to census population size (Nc/Ne), mean return day, and mean reproductive lifespan. Output parameters were estimated as the mean of 100 model iterations and are bounded by 95% confidence intervals. Comparisons within the 10th generation are provided in the supplemental materials (Figure S4).

We investigated how the magnitude of negative phenotypic correlation (⍴) between return timing and RLS affected demography and evolution (simulations with positive correlations are reported in Figure S2). In simulations with no correlation between traits, population sizes grew in 10 generations from 500 to 1514 individuals, 95% CI [1313, 1715]; however, in simulations with higher degrees of correlation between traits (i.e., −0.6), population sizes declined from 500 to 256 individuals, 95% CI [207, 305] over the same time period (Figure 3a). From this result, we concluded that greater correlation magnitudes (i.e., more negative correlations) between traits resulted in reduced population growth. Examination of the effect of ⍴ on Ne/Nc ratio showed that when traits were uncorrelated, Ne/Nc was higher (0.66, 95% CI [0.65–0.67] after 10 generations) than when traits were highly negatively correlated (0.56, 95% CI [0.54, 0.58] after 10 generations; Figure 3d). The relationship between ⍴ and Ne/Nc also appeared non‐linear, with a large difference in Ne/Nc between ⍴ values of −0.3 and −0.6, and a small difference in Ne/Nc between ⍴ values of 0 and −0.3. There was no difference between mean return day values in simulations with different ⍴ (Figure 3g), indicating no relationship between ⍴ and the evolution of return day. However, simulations with a high degree of correlation (i.e., ⍴ = −0.6) resulted in the evolution of a longer average RLS (after 10 generations, mean RLS = 7.83 days, 95% CI [7.95, 7.71]) compared to simulations with no correlation between traits (7.27 days, 95% CI [7.20, 7.34]; Figure 3j).

### 3.3 Experiment 3: Interannual environmental variability

We next investigated how variation in interannual ecological optima affected population dynamics. In simulations where the optimal return day was allowed to vary greatly between years (*σ* ^2^_θ Return Day_ = 30) population sizes grew only slightly from 500 to 590 individuals 95% CI, [495, 685] in 10 generations; whereas in simulations with low *σ* ^2^_θ Return Day_ (i.e., 10) population sizes grew to 1788 individuals, 95% CI [1622, 1954] (Figure 3b). This result demonstrated a negative relationship between *σ* ^2^_θ Return Day_ and population growth rate. In addition, this relationship was non‐linear, as the difference in Nc between simulations with *σ* ^2^_θ Return Day_ values of 10 and 20 was much greater than the difference between simulations with *σ* ^2^_θ Return Day_ values of 20 and 30.

The effect of environmental variability on evolutionary dynamics was more subtle. Simulations with more stable interannual environmental regimes (*σ* ^2^_θ Return Day_ = 10) resulted in only slightly higher Ne/Nc ratios (i.e., 0.65, 95% CI [0.64, 0.66] after 10 generations) than simulations with more variable environments (i.e., 0.59, 95% CI, [0.57, 0.61] after 10 generations when *σ* ^2^_θ Return Day_ = 30; Figure 3e). Furthermore, more variable simulations saw the evolution of marginally later mean return days (10.94 days, 95% CI [10.49, 11.39] when *σ* ^2^_θ Return Day_ = 30 compared to 10.18 days, 95% CI [9.87, 10.49] when *σ* ^2^_θ Return Day_ = 10; Figure 3h). Yet no relationship was found between *σ* ^2^_θ Return Day_ and the evolution of mean RLS (Figure 3k).

### 3.4 Experiment 4: Strength of selection

We lastly examined the relationship between population dynamics and the intensity of stabilizing selection on return timing (1/**ω**). Simulations with weak selection regimes (**ω** = 3) had populations which grew nearly 1000% within 10 generations (500 to 4528 individuals, 95% CI [4041, 5015]), whereas simulations with strong selection regimes (**ω** = 1) showed populations which declined to near extinction (mean Nc = 79, 95% CI [55, 103]) (Figure 3c). Population sizes remained relatively stable in simulations with moderate selection regimes (**ω** = 2), growing from 500 to 866 individuals, 95% CI [750, 982] in 10 generations. From these results we concluded that **ω** had a strong positive correlation with Nc. Selection intensity also had a strong effect on Ne/Nc ratio: simulations with weak selection had greater Ne/Nc ratios (0.71, 95% CI [0.70, 00.72] after 10 generations when **ω** = 3; 0.46, 95% CI [0.39, 0.53] when **ω** = 1; Figure 3f). This result indicated a strong positive relationship between **ω** and Ne/Nc. No relationship was observed between **ω** and the evolution of mean return day (Figure 3i). However, when selection was weak, populations evolved to have a longer average RLS (7.91 days, 95% CI [7.84, 7.98] when **ω** = 3; 6.85 days, 95% CI [6.61, 7.09] when **ω** = 1), indicating a positive correlation between **ω** and mean RLS (Figure 3l).

## 4 DISCUSSION

Here we introduced a modular framework for modeling population dynamics that incorporated eco‐evolutionary principles and included temporally distributed assortative mating, closely resembling that of many aggregative breeders. The primary benefits of this framework, compared to other existing population dynamics models, are that it is both mechanistic and predictive, allowing for flexible hypothesis testing of proposed management scenarios. Specifically, the model can be parameterized with empirical values from specific study systems and used to predict demo‐evolutionary outcomes of changes to input parameters. The utility and flexibility of this framework was demonstrated through a series of simulation experiments to examine the effects of four common population dynamics on demography and evolution in simulated salmonid populations: (1) temporal assortative mating dynamics, (2) correlation between fitness‐linked traits, (3) interannual environmental variability, and (4) strength of selection. We found that temporal assortative mating had an overall positive effect on population growth. Unexpectedly, the phenotypic variance in return day – which was used as a proxy for the magnitude of assortative mating regime – also had a positive relationship with population growth. We further found that increasing the magnitude of trait correlations, environmental variability, and strength of selection all had negative effects on population growth. These findings demonstrate how our model performs as expected for known population dynamics, while allowing mechanistic exploration of how proposed management strategies might affect demography and evolution in wild populations.

### 4.1 Explanation and implications of assortative mating simulations

We examined the effects of assortative mating on demography by either preventing or allowing matings to occur between pairs of individuals who were not present in the stream at the same time. The results demonstrated population growth under assortative mating but declines under random mating. Higher population growth under assortative mating can be explained by increased correlation of breeding values between mates, a byproduct of correlated phenotypes between mates under assortative mating regimes (Felsenstein, 1981). As fitness in this model was the product of the expected fitness of dams and sires, mating pairs with high breeding values (i.e., individuals near the optimal trait value, θ_return day_) resulted in higher mean fitness (i.e., population growth rate) than when populations mated randomly. Examined from a different perspective, random mating also limited the response to selection, thus preventing adaptation to fitness optima and driving population declines. These results highlight the possible role of assortative mating in maintaining population adaptive capacity and resilience. In the first experiment, interannual environmental variation was held constant at an intermediate value, and mean return days evolved much closer to the mean optimal return day ($\bar{\theta }$_Return Day_) in populations with assortative mating than in populations under random mating. In other words, populations with assortative mating were able to better track a variable fitness optimum (further demonstrated in Figures S3 and S5).

These results complement those in Godineau et al. (2022) who, using a polygenic model with similar eco‐evolutionary parameters to our model, showed how assortative mating may increase adaptive capacity for plant flowering phenology. Unlike our model, the model in Godineau et al. (2022) assumes a constant population size, and they found no effect of assortative mating on adaptation to fluctuating selection. However, the authors did find a positive effect of assortative mating on adaptation to an environment with an increasing optimum over time, which we did not examine here. By contrast, in our model, the phenotypic variance was constant but the population size was allowed to vary, and we found a positive effect of assortative mating on adaptive capacity in response to environmental fluctuations with no trend. We thus conclude that there may be a twofold advantage of assortative mating on both genetic variance and demography in a changing environment (defined by both fluctuations and a trend). Our results further confirm that phenological assortative mating dynamics are important to the adaptive capacity and resilience of populations.

Here, we varied the phenotypic variance in return day (*σ* ^2^_Return Day_), which had an inverse relationship with heritability and therefore an inverse relationship with the magnitude of assortative mating. We originally expected that higher heritability values would increase the magnitude of assortative mating, where increasing the correlation of breeding values between mates would result in higher population growth rates. Yet we observed the opposite trend: simulations with low phenotypic variance (i.e., high heritability and magnitude of assortative mating) had reduced recruitment. Upon further examination, we attributed this result to the effect of phenotypic variance on selection, whereby increased phenotypic variance resulted in higher expected fitness values for a given trait value (this relationship is further detailed in the Figures S1–S5). Increasing heritability did have a small effect on population genetic diversity, demonstrated by the Ne/Nc ratio: increasing *σ* ^2^_Return Day_ (i.e., decreasing heritability and the strength of assortative mating) resulted in a slightly increased Ne/Nc ratio. In other words, populations with a weaker magnitude of assortative mating more closely resembled an idealized Wright‐Fisher population (randomly mating). In addition, because the Ne/Nc ratio was lower under stronger assortative mating regimes, we concluded that genetic diversity is also likely lower when the magnitude of assortative mating increases (i.e., lower *σ* ^2^_Return Day_), which confirms findings by Godineau et al. (2022). However, the indirect effect of increased heritability on recruitment was substantially weaker than the direct effect of decreased phenotypic variation. This overall positive relationship between phenotypic variation and recruitment highlights the relative importance of phenotypic variation to recruitment and population growth in natural populations.

### 4.2 Trait correlations hinder evolution to fitness optima

Varying the negative correlation (⍴) between return day and RLS revealed a negative relationship between the magnitude of this correlation and population growth. This relationship can be explained by the effect of ⍴ on the evolution of mean trait values. Return day evolved to a population mean close to $\bar{\theta }$_Return Day_ (10 days) and was independent of ⍴. However, there was an effect of ⍴ on RLS. The mean optimal RLS ($\bar{\theta }$_RLS_) was held constant at 5 days, but mean RLS did not evolve near this optimum across simulations. This result can be explained by an internal model mechanic within the inheritance module whereby return day is drawn before RLS from a univariate Gaussian distribution. Then, RLS is drawn from a second Gaussian distribution with a mean and variance conditional on the value drawn for return day. Therefore, return day is not conditional on RLS, but RLS is conditional on return day, by the magnitude of ⍴. This mechanic resulted in the evolution of longer average RLS in simulations with greater correlation between traits (more negative ⍴), because the evolution of RLS was constrained by ⍴ such that the populations could not evolve to $\bar{\theta }$ when RLS and entry day were phenotypically correlated. This mechanic reduced mean fitness and population growth when correlations were greater. Notably, RLS was not able to evolve to its phenotypic optimum (even when uncorrelated; Figure S3), because the truncated distributions used to draw traits resulted in a shift in mean phenotype. Truncated distributions were biologically relevant for the phenotypes examined here, but future model applications should consider whether to apply truncated or true Gaussian distributions when drawing trait values.

A fundamental assumption of this modeling framework is that stabilizing selection acts on a fitness landscape with a single Gaussian fitness peak. Although this is a common assumption in many similar eco‐evolutionary models (i.e., Lin et al., 2017; Yeakel et al., 2018) and is consistent with many empirically observed patterns of fitness‐linked traits in anadromous salmonids (i.e., Lin et al., 2016), Gaussian selection is not an accurate representation of selection in many natural populations (Huisman & Tufto, 2012; Laughlin, 2018). For example, negative correlations between RLS and return day in Sockeye salmon (*Oncorhynchus nerka*) may exhibit an adaptive tradeoff and therefore convey similar fitness benefits to early returning individuals with long RLS and later returning individuals with short RLS (Doctor & Quinn, 2009; Lin et al., 2016). Therefore, we recommend caution when imposing correlations on traits in future applications of this model, without applying an appropriately complex fitness landscape. Yet, expansion of the predictive framework outlined by Lande and Arnold (1983) into multi‐modal and multivariate fitness landscapes remains a challenge in applications of quantitative genetic theory.

### 4.3 Simulation outcomes for environmental variation and selection allow for empirical parameterization

A primary prediction of many climate change studies is that local environmental and ecological regimes will likely become more variable. For example, in many parts of the northern hemisphere, interannual temperature variation has increased by more than 25% since 1980 (Huntingford et al., 2013). We examined the effects of such increased environmental variability by shifting the fitness peak in our simulations after each generation, such that populations were constantly chasing a moving optimum. Increasing the magnitude of interannual variability hindered the evolution of traits to their optima, which drove population declines when variability was very high and would have resulted in extinction if simulations were allowed to run for additional generations or were exposed to stronger selection regimes. This result broadly agrees with established theory that shifting environmental regimes can drive declines in population mean fitness (Bürger, 1999; Bürger & Lynch, 1995; Charlesworth, 1993; Kopp & Matuszewski, 2014; Lynch, 1991). This result demonstrates the utility of our model to examine the demographic and evolutionary consequences of environmental variability in specific empirical study systems (e.g., under climate change scenarios). When parameterized with empirically measured values, our framework may be applied to specific study systems to project future changes. For example in flowering plants, phenological traits such as flowering date and maturation timing of seeds have garnered recent attention in studies of how these traits will respond to climate change scenarios (i.e., Duputié et al., 2015; Gauzere et al., 2020; Godineau et al., 2022). These phenological traits are somewhat analogous to the salmonid traits of return timing and reproductive lifespan examined here, and we emphasize that our modeling framework may prove useful in diverse study systems.

Similarly, the result that weaker stabilizing selection resulted in increased population growth also conforms well to theoretical expectations (Lande, 1976). The strength of selection on various traits is a central quantifiable measurement that is reported in many studies of empirical, natural populations. As such, our model presents a mechanistic framework that allows for parameterization with empirical measurements of selection magnitude and direction on diverse traits and in diverse taxa. However, as noted when discussing trait correlations, our model is somewhat limited by the assumption that selection acts on a single Gaussian fitness peak. For this model to be best representative of specific study populations, there is a need to incorporate more complex selection regimes by predicting expected fitness values on more dynamic fitness landscapes (i.e., with multiple fitness peaks and troughs; Huisman & Tufto, 2012). In addition, most variation in salmonid population growth is typically attributed to variation in reproductive success during freshwater residency or at‐sea mortality (Koch & Narum, 2021), not as a direct result of selection on traits during reproduction (as modeled here). Thus, model dynamics should be considered as only one of many possible sources of variation in population growth in specific study systems.

## 5 CONCLUSIONS

There is greater awareness of the role that eco‐evolutionary processes contribute to population dynamics (Palkovacs & Hendry, 2010), and an urgent need from decision makers for tools to understand the consequences of environmental perturbations on natural populations. Across many species, climate change is one of the most obvious drivers that interacts with timing and assortative mating to shape individual fitness (Anderson et al., 2016; Tillotson et al., 2019). Results from our simulations highlight that assuming mating is random when it is non‐random may underestimate population resilience to perturbations and overestimate the impacts of those perturbations on demography. One opportunity for this work to inform future management at the population level is through strengthened ties to empirical data and monitoring programs. Previous research has highlighted the need to collect population‐level data on processes of dispersal, adaptation, and genetic variation (Eldridge et al., 2010; Lin et al., 2017; Pelletier et al., 2009; Zheng et al., 2009). For other aggregate breeders, including some mammal or bird species, or sessile species such as plants, collecting additional data to inform mate choice may be beneficial (Dittrich et al., 2018; Wang et al., 2019). Even in well studied species such as salmonids, the effects of mate choice on LRS and demography remains unclear (Auld et al., 2019). These additional data sources may be linked to our existing framework via statistical models (e.g., generalized linear models), and both model and parameter uncertainty may be incorporated as additional sources of variation.

We expect future research will also improve the statistical modeling underpinning our framework. One potential line of future work is to expand on the assumption of Gaussian selection curves and include a suite of other parametric approaches that allow for greater extremes (e.g., multivariate Student‐t distributions). A second potential line of research is to investigate the strength or direction of selection on assortative mating in space or time; building these trend parameters into our simulation framework is expected to improve predictions under future scenarios (such as a warming environment). A third avenue of research is extending the general framework to multiple populations and to a spatial dimension – this may be particularly useful for species such as salmonids, that often exist as a metapopulation in both space and time or have overlapping sub‐populations of wild and hatchery components. Finally, we made a number of specific assumptions to tailor our framework to a generalized salmonid life history, and future model applications will serve to elucidate how results may change when specific model mechanics change to match different life histories. While specific applications of our framework are expected to vary species to species, additional customization of this model will improve inference about eco‐evolutionary processes in natural populations and provide better guidance to natural resource managers in a rapidly changing world.

## CONFLICT OF INTEREST

The authors report no conflicts of interest.

## DATA AVAILABLITY STATEMENT

All code and data for this study are provided in our publicly available Github repository linked here: https://github.com/SMay1/Assortative_Mating_QG_IBM and Zenodo linked here: https://zenodo.org/record/6981706

## Supporting information


Figures S1–S5.
