# Supplementary material for: Assortative mating for reproductive timing affects population recruitment and resilience in a quantitative genetic model
Source: Evol Appl. 2023 Jan 20;16(3):657–72. doi: 10.1111/eva.13524 (PMC10033844; doi:10.1111/eva.13524)
Supplement: Supplementary file 1 — Figures S1–S5. [file EVA-16-657-s001.docx]

**Supplemental Information:**


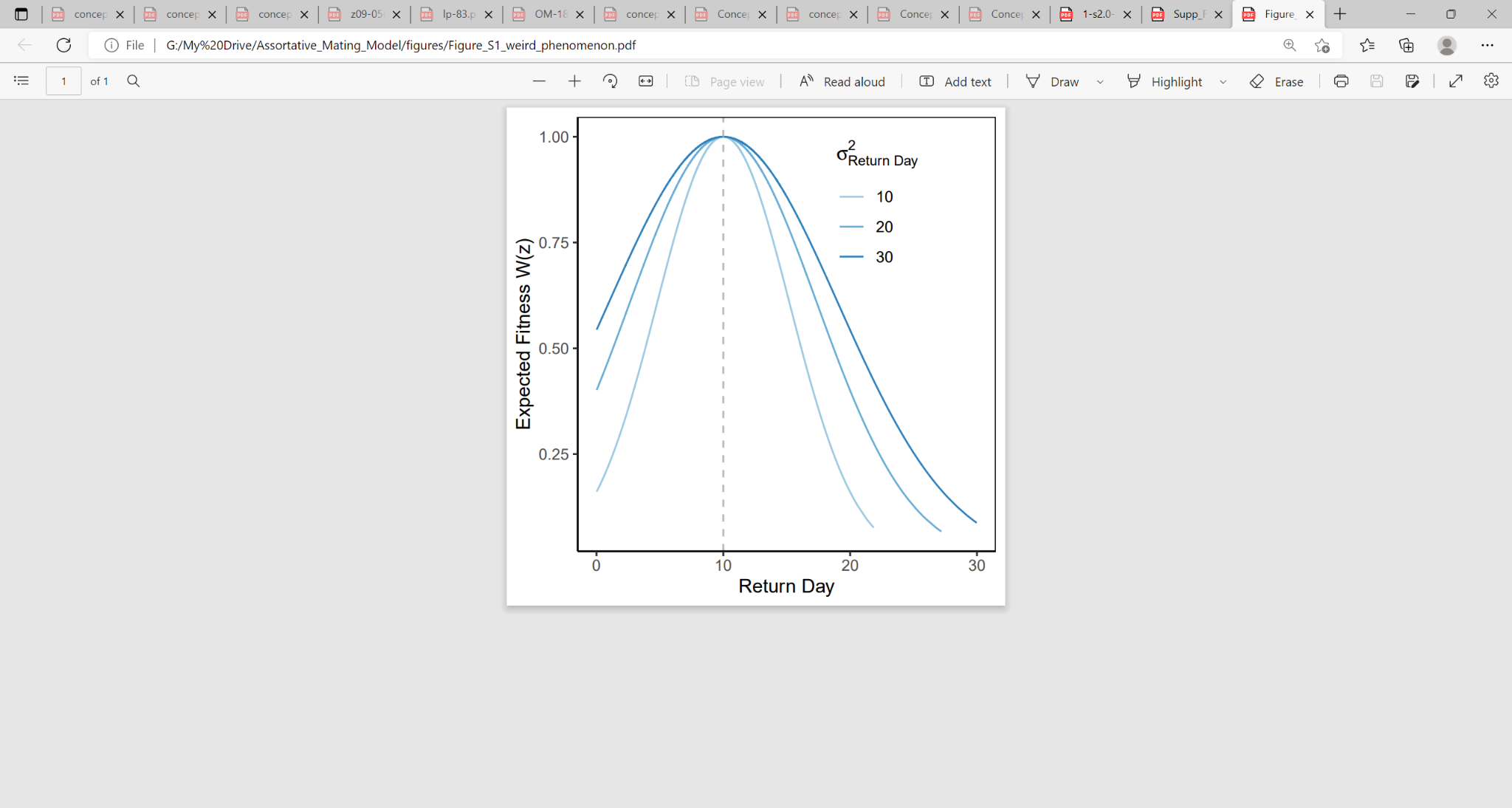


Supplemental Figure 1. The relationship between return day (x-axis) and expected fitness (y-axis) for three different values of phenotypic variance in return day (σ²_Return Day_). When σ²_Return Day_ = 30, an individual with a given trait value (return day) will tend to have more offspring than when σ²_Return Day_ = 10. This figure was produced by varying σ²_Return Day_ for three ‘populations’ of 100,000 individuals, drawing from the phenotypic distributions detailed in the main text, and quantifying W(z) values using Equation 1 (in standard, not matrix, notation). In standard notation, **⍵** is expressed as a scalar number of phenotypic standard deviations away from **θ.** The value for **⍵** in Equation 1 is thus dependent on the phenotypic variance in the population, which is sometimes referred to as “variance available to selection.” This standard notation is applicable when omega is constant (i.e., when **G**, **R**, and **P** are also constant), which is a key assumption in our paper. This phenomenon explains how phenotypic variance can affect observed reproductive success values and population growth rates under both assortative and random mating systems.


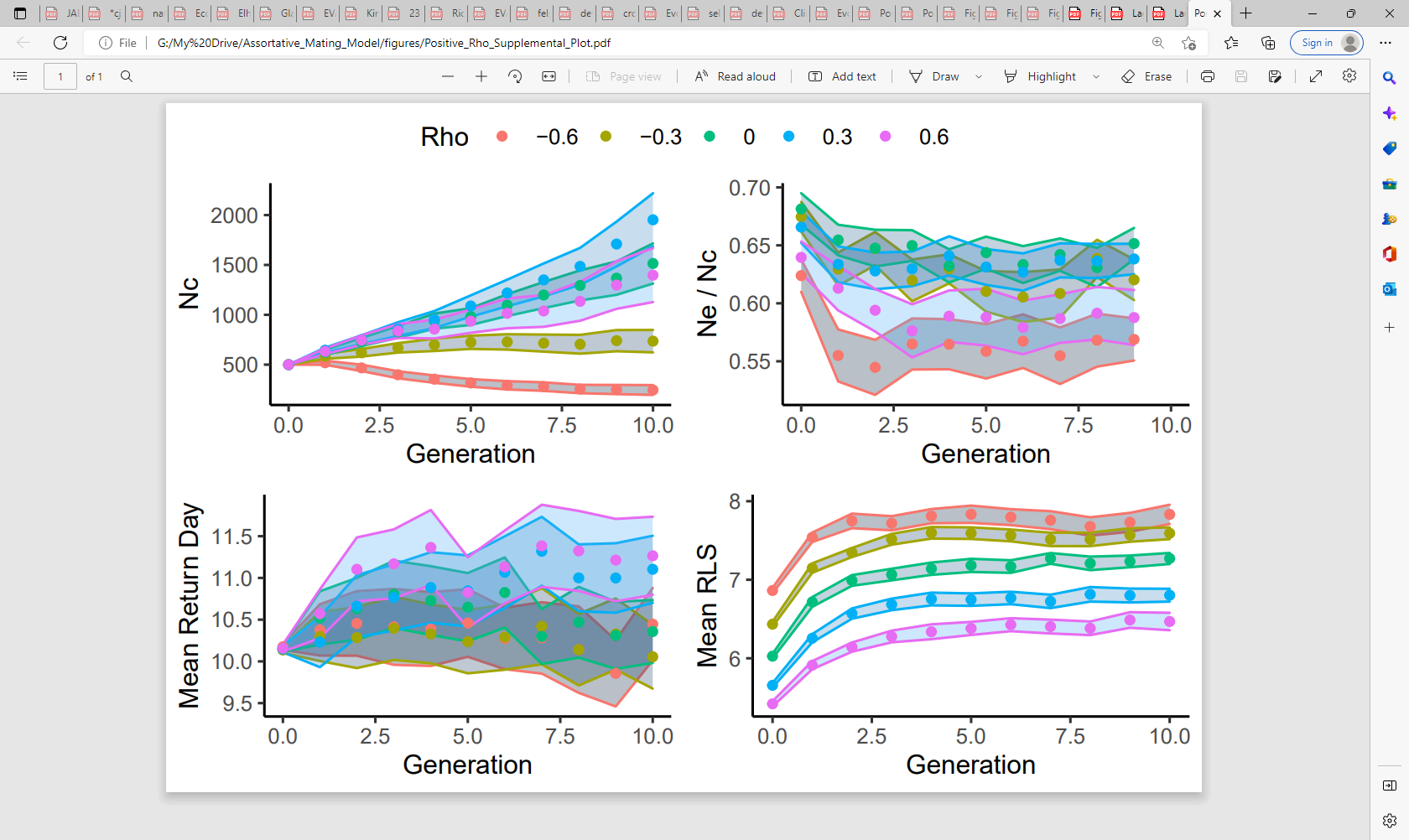


Supplemental Figure 2. Demographic output parameters (y-axes) over 10 generations (x-axes) for different values of rho (colors) while all other parameters were constant. Output parameters of interest (points) were census population size (Nc), ratio of effective to census population size (Nc/Ne), mean return day, and mean reproductive lifespan (RLS). Output parameters were estimated as the mean of 100 model iterations and are bounded by 95% confidence intervals. These outputs are identical to those presented in Figure 3 (orange plots), with the addition of positive rho values of 0.3 and 0.6. These results demonstrate the relatively minor effect of positive correlations between return day and reproductive lifespan (compared to no correlation, rho = 0) on census size, effective population size, and the evolution of mean return day. Positive rho values enabled the evolution of RLS closer to its optimum ($\bar{\theta}$_RLS_ = 5), because rho affects the mean trait values of the truncated bivariate normal distribution (from which RLS is drawn). We note that the direction and magnitude of rho, and its effects on demographic and evolutionary parameters, may differ for different traits of interest (i.e., body size or coloration), different trait distributions (i.e., Gaussian instead of truncated-Gaussian), and in different study systems.


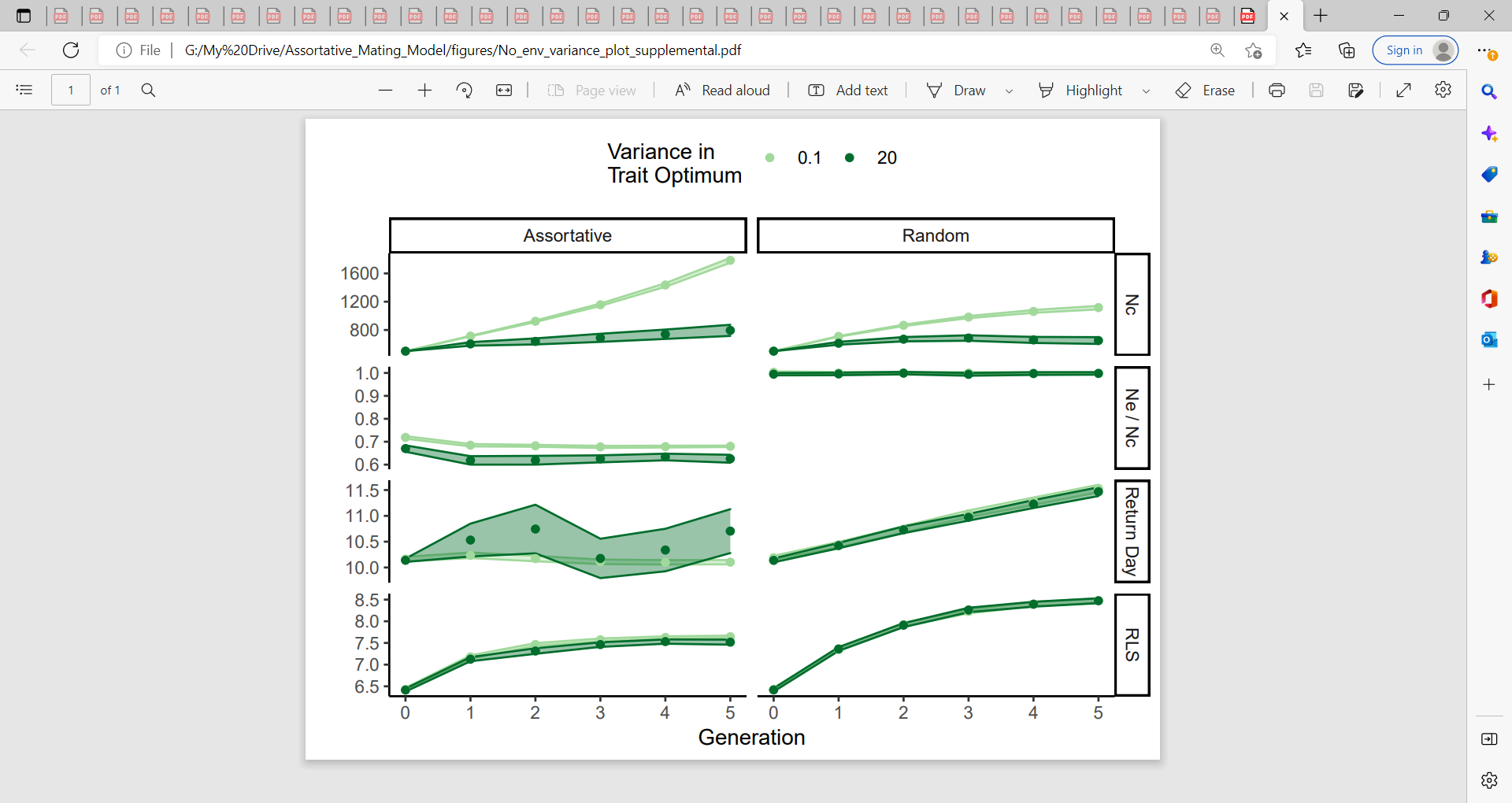


Supplemental Figure 3. Demographic output parameters (y-axes) over 5 generations (x-axes) for two different values of variance in optimal return day (σ^2^_θ_ _Return Day_; shades), while all other parameters were constant as in previous analyses. Specific to this analysis, the variance in optimal RLS (σ^2^_θ_ _RLS_) was held constant at zero. Output parameters of interest (points) were census population size (Nc), ratio of effective to census population size (Nc/Ne), mean return day, and mean reproductive lifespan (RLS). Output parameters were estimated as the mean of 100 model iterations and are bounded by 95% confidence intervals. In this analysis, σ^2^_θ_ _Return Day_ values of 20 (used in other analyses throughout the main text) were compared to extremely small values of 0.1 (close to zero). Values of 0.1 were used instead of zero, because values of zero for both σ^2^_θ_ _Return Day_ and σ^2^_θ_ _RLS_ would result in individuals with W(z) values equal to 1. These W(z) values are used as a probability vector to draw from a Poisson distribution, and probability values equal to 1 result in individuals with an infinite number of offspring. Nevertheless, these results demonstrate how, under random mating, the variance in trait optimum does not affect the evolution of traits. However, populations will be near the optimum more often when σ^2^_θ_ _Return Day_ is low, resulting in greater population growth. Under random mating, traits evolve towards their equilibrium values, which is driven exclusively by the constant phenotypic variance in traits and the truncated distributions from which the traits are drawn. On the other hand, under assortative mating, populations are able to evolve very near to the optimal return day, because mating pairs close to the optimum will contribute disproportionately more offspring to the next generation. When environmental variation is greater, there is greater variation in mean return day, but it is still quite close to the optimum. In effect, these results show how assortative mating bolsters population stability, by perpetuating populations that are very near the phenotypic optimum, even when it is variable. RLS under assortative mating is still dependent (and correlated with) return day (a product of our model which is described in the main text), which prevents it from reaching its true optimum (5 days); however, RLS still evolves closer to the optimum under assortative mating than it does under random mating.


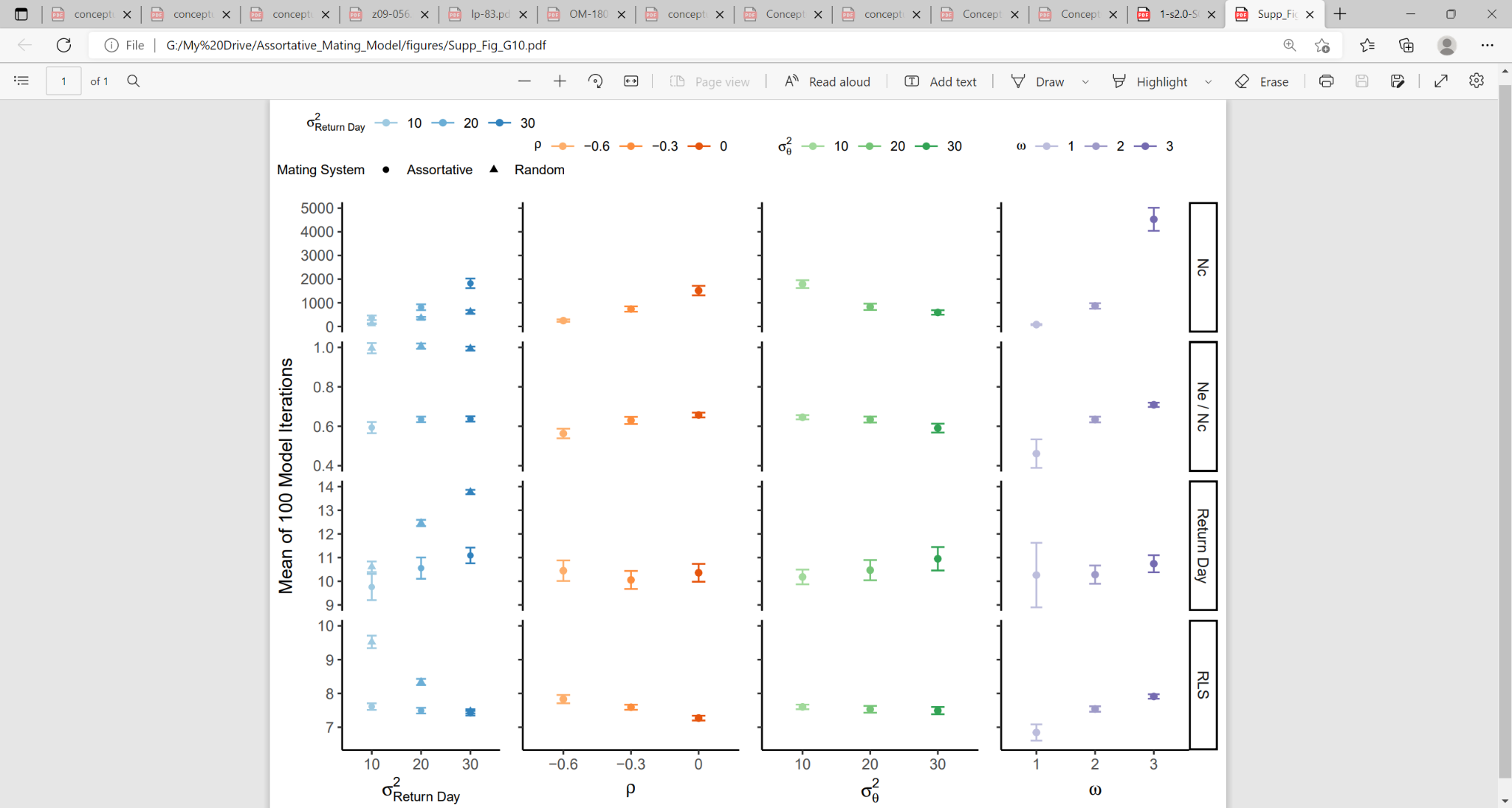


Supplemental Figure 4. Demographic output parameters for the 10th generation (y-axes) of four experiments (colors). Each experiment consisted of three variations of a single input parameter (x-axes, shades), while all other parameters were constant as described in the text. The input parameters of interest were phenotypic variance in return day (σ²_Return Day_; blues), phenotypic correlation between return day and reproductive lifespan (⍴; oranges), variance in optimum return day (σ²_θ Return Day_; greens), and strength of selection regime (**ω**; purples). Output parameters of interest (points) from top to bottom were census population size (Nc), ratio of effective to census population size (Nc/Ne), mean return day, and mean reproductive lifespan (RLS). Output parameters were estimated as the mean of 100 model iterations and are bounded by 95% confidence intervals. This Fig. uses the same data as in Figs. 2-3, in the main text, but only shows the last generation (F10). Color shades here are redundant with x-axis labels, but correspond with colors in Figs. 2-3, in the main text.


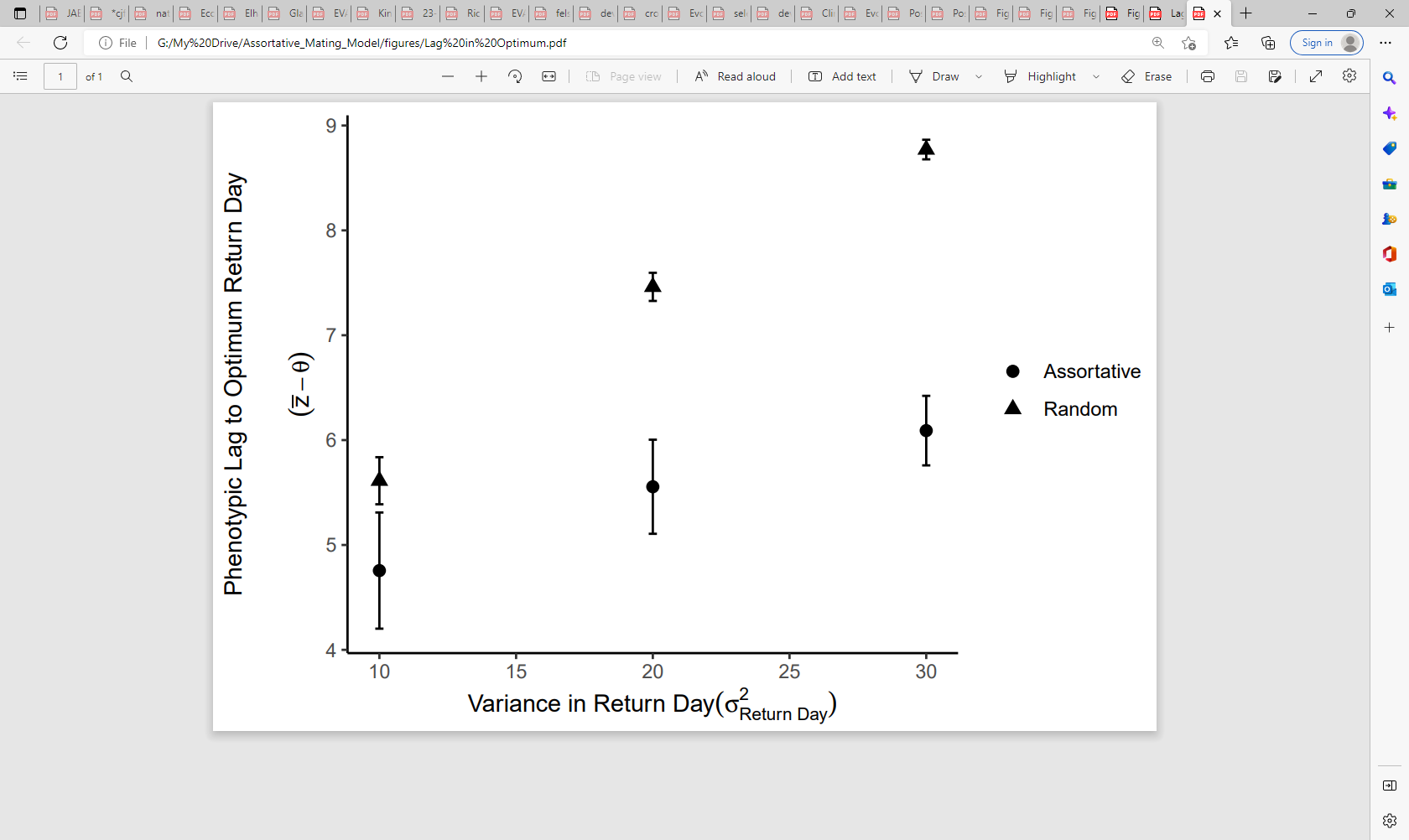


Supplemental Figure 5. Lag of the population phenotypic mean ($\bar{z}$) to the phenotypic optimum (θ) for return day after 10 generations of simulation iterations. Lag values (y-axis) are given for three separate simulations using different values for variance in return day (x-axis) and for both assortative (points) and random (triangles) mating systems. Lag values are given as a mean of 100 model iterations for each unique set of input parameters bounded by 95% confidence limits. These results demonstrate how populations with assortative mating systems better track the moving phenotypic optimum, which moves within each generation about an overall mean ($\bar{\theta}$_Return Day_ = 5 for these simulations) according to a predetermined variance (σ²_Return Day_ = 20 for these simulations).
